# Transition to adult care in youth with type 1 diabetes: follow-up continuity, complications, and metabolic control

**DOI:** 10.1530/EC-25-0495

**Published:** 2025-11-06

**Authors:** Uğur Cem Yılmaz, Melih Bektaş, Yiğit Özel, Günay Demir, Deniz Özalp Kızılay, Şükran Darcan, Samim Özen, Vildan Özkan Derviş, Ilgın Yıldırım Şimşir, Damla Gökşen

**Affiliations:** ^1^ Division of Pediatric Endocrinology and Diabetes, Ege University Faculty of Medicine, İzmir, Turkey; ^2^ Division of Endocrinology, Ege University Faculty of Medicine, İzmir, Turkey; ^3^ Department of Pediatrics, Ege University Faculty of Medicine, İzmir, Turkey

**Keywords:** type 1 diabetes, transition to adult care, metabolic control, follow-up continuity, HbA1c levels, complications, transition clinic, multidisciplinary support, diabetes management

## Abstract

**Introduction:**

The transition from pediatric to adult care in individuals with type 1 diabetes (T1D) often presents significant challenges, including disruptions in follow-up continuity and metabolic control.

**Objective:**

The aim of this study is to assess healthcare utilization, follow-up continuity, metabolic control (HbA1c levels), and the development of complications after transition to adult care within a transition clinic setting.

**Methods:**

A retrospective analysis was performed on 118 individuals with T1D who transitioned to adult care. Demographic data, along with pre- and post-transition HbA1c levels, chronic complications, treatment modifications, and follow-up attendance, were collected and analyzed.

**Results:**

Of the 118 participants, 67% (*n* = 80) transitioned through the transition council, with 62.5% (*n* = 50) maintaining regular follow-up in adult care. However, 27.5% (*n* = 30) experienced follow-up discontinuity. The mean HbA1c in the last year of pediatric care was 7.95 ± 1.27%, which slightly decreased to 7.73 ± 1.17% in the first year of adult care and remained stable at 7.74 ± 1.17% in the second year. Complication rates increased from 18% pre-transition to 26% during adult follow-up.

**Conclusion:**

Despite the challenges faced during the transition, transition clinics play a crucial role in supporting follow-up continuity and improving metabolic control. Multidisciplinary care and individualized treatment modifications are essential in reducing complication risks. Future research should include larger sample sizes to better address long-term health outcomes and optimize the transition process.

## Introduction

Type 1 diabetes (T1D) is one of the most prevalent chronic endocrine disorders of childhood and adolescence, requiring lifelong monitoring and management. Effective T1D care involves a multidisciplinary approach (1). In pediatric care, individuals are closely followed by a team of pediatric endocrinologists, dietitians, nurses, and psychologists. However, during the transition to adult care, follow-up continuity may be disrupted, leading to compromised metabolic control (2). Structured and planned transitions are recommended to minimize adverse outcomes (3).

Transitioning to adult care is a complex and multifaceted process. Individuals may face reduced healthcare utilization, adaptation challenges, and psychosocial issues (4). Weissberg-Benchell *et al.* (5) found that inadequate planning negatively impacts health outcomes and reduces follow-up continuity (5). Therefore, structured guidance on self-management and healthcare access is crucial. The transition age should also consider the individual’s psychosocial maturity (3). Randomized studies show that appointment coordination support improves clinic attendance and treatment adherence (3). Transition clinics play a significant role in facilitating this process. Collaboration between pediatric and adult care teams through individualized transition plans enhances follow-up continuity and patient satisfaction (6, 7). Recommended strategies include appointing a transition coordinator, documenting a written care plan, and maintaining regular appointment follow-ups (3).

Despite these findings, there is limited information on long-term follow-up outcomes and complication rates post-transition, necessitating further research (8). This study evaluates healthcare utilization and follow-up continuity in T1D patients within a transition clinic and aims to provide insights to optimize transition processes and metabolic control.

## Materials and methods

This study retrospectively analyzed the transition process of 118 individuals diagnosed with type 1 diabetes (T1D) to adult care at the transition clinic in a tertiary university clinic. A structured transition process is implemented in this clinic, where a multidisciplinary transition committee meeting is held, attended by pediatric and adult endocrinologists, diabetes nurses, and dietitians. This committee convenes monthly with the participation of all team members. During these meetings, individuals undergoing transition receive a comprehensive assessment, case discussions are conducted without the physical presence of the individuals, and a structured transition plan is developed. After the decision for transition was made during the joint pediatric–adult meeting and the patient was handed over, the adult endocrinology team directly contacted the individual to arrange the first appointment, and the pediatric team subsequently confirmed with the patient that this contact had been established. In this study, ‘discontinued follow-up at our center’ was defined as not returning to our adult endocrinology clinic for ≥12 months after the first visit or never attending. At the end of the process, to ensure continuity of care, appointments with adult endocrinology services are scheduled, facilitating a seamless transition to adult diabetes care.

Demographic data, including age, sex, diabetes duration, and history of complications, were collected. Differences between pre- and post-transition follow-up processes were evaluated through a review of clinical records.

### Data collection process

Data were gathered from electronic medical records and written documents. Variables such as HbA1c levels, complication rates, therapeutic regimen modifications, and adherence to regular follow-up appointments were examined. In addition, participants’ healthcare utilization was assessed.

### Statistical analysis

Data were analyzed using SPSS version 25.0. Continuous variables were expressed as mean ± standard deviation, while categorical variables were presented as percentages (%). Paired *t*-tests and chi-square tests were used to compare pre- and post-transition data. For non-normally distributed variables, the Wilcoxon signed-rank test and Mann–Whitney U test were applied. A *P*-value of <0.05 was considered statistically significant.

## Results

### Case demographics and care continuity preferences

Between 2010 and 2024, a total of 118 individuals diagnosed with type 1 diabetes (T1D) who transitioned from pediatric to adult endocrinology care were evaluated within the transition committee. Of these participants, 52.5% (*n* = 62) were female, and 47.5% (*n* = 56) were male. At the initial assessment, 38 individuals expressed a preference to continue follow-up at other institutions. The remaining 67.8% (*n* = 80) were enrolled in follow-up at the same university adult endocrinology unit. Among these individuals, 50 (62.5%) attended their first adult endocrinology appointment within the first year post-transition and maintained regular follow-up at intervals of no more than 6 months. However, 30 individuals (37.5%) gradually discontinued follow-up after their initial visit and were subsequently lost to structured care (Table 1).

**Table 1:** Transition process data for individuals with type 1 diabetes from pediatric to adult care.

Feature	Values
Number of individuals transitioned	118
Gender	Female: 62 (52.5%); male: 56 (47.5%)
Individuals presenting to adult endocrinology	80 (67.8%)
Individuals requesting follow-up at different centers (initial visit)	38
Individuals continuing follow-up in adult care	50/80 (62.5%)
Individuals who discontinued follow-up	30/80 (37.5%)
Transition age (years)	Mean: 21.38 ± 2.75
Duration of diabetes (years)	Mean: 12.16 ± 4.32
Time from transition to first follow-up (years)	Mean: 0.33 ± 0.29
Adult follow-up duration (years)	Mean: 2.58 ± 2.22
Follow-up frequency (visits per year)	Pediatric: 3.19; adult: 4.27
Mean HbA1c	Last pediatric year: 7.95% ± 1.27; first adult year: 7.73% ± 1.17; second adult year: 7.74% ± 1.17
Dietitian support	Pre-transition: 100%; post-transition: 70%
Mean BMI	Pediatric last visit: 22.67 ± 3.47; adult last visit: 23.68 ± 3.84
Psychiatric follow-up	Pediatric: 36%; adult: 26%
Insulin dose (U/kg)	Pediatric: 0.77 ± 0.20; adult: 1.36 ± 0.54
Basal insulin usage (%)	Pediatric: 44.65 ± 11.75; adult: 45.57 ± 9.97
Types and rates of treatment adjustments	U100 to U300 glargine: 37% (*n* = 10); metformin addition: 19% (*n* = 5); detemir to U300 glargine: 19% (*n* = 5); MDI to pump therapy: 15% (*n* = 4); pump to MDI: 11% (*n* = 3)
New complications developed	Pediatric follow-up: 18%; adult follow-up: 26%
Types of complications	Nephropathy: pediatric 4; adult 3; peripheral neuropathy: pediatric 3; adult 5; retinopathy: pediatric 2; adult 5

BMI, body mass index.

### Transition age and continuity of care

The average age at transition was 21.38 ± 2.75 years, with a mean diabetes duration of 12.16 ± 4.32 years. The mean time from transition to the first follow-up visit was calculated as 0.33 ± 0.29 years (min–max: 0–0.99 years), while the average duration of adult care follow-up was 2.58 ± 2.22 years. All comparative analyses regarding follow-up frequency before and after transition were performed exclusively for individuals who maintained regular follow-up in adult care. Follow-up frequency in adult care was significantly higher than in pediatric care (mean: 4.27 visits/year vs 3.19 visits/year, *P* = 0.02) (Table 1). In addition, care continuity significantly increased as the age at transition increased (*P* = 0.02). However, among the 50 individuals who maintained follow-up, 34% (*n* = 17) discontinued care after an average of 2.58 ± 2.19 years. Individuals with no recorded follow-up visits in the last 6 months were considered to have discontinued care.

### Glycemic control, nutritional counseling, and body mass index (BMI)

**Figure 1:**
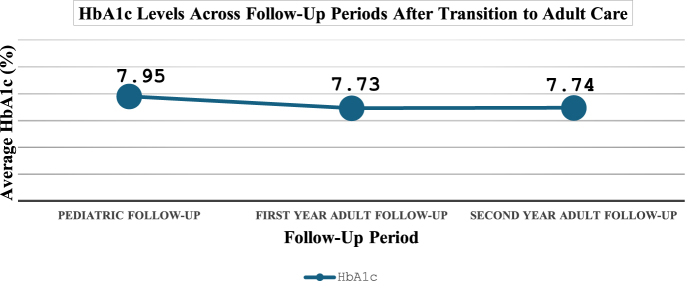
Mean HbA1c (%) values are presented for three consecutive follow-up periods: pediatric follow-up, first year of adult follow-up, and second year of adult follow-up. Data are expressed as mean ± SD. The vertical axis represents HbA1c (%), and the horizontal axis indicates the respective follow-up periods.

In the final year of pediatric follow-up, the mean HbA1c level was 7.95 ± 1.27%. This value decreased slightly to 7.73 ± 1.17% in the first year of adult care and remained stable at 7.74 ± 1.17% in the second year (*P* = 0.42) (Fig. 1). Before transition, all individuals received nutritional counseling and were practicing carbohydrate counting, which decreased to 70% post-transition. When comparing BMI between the final pediatric and adult follow-up groups, the mean BMI increased from 22.67 ± 3.47 to 23.68 ± 3.84 (*P* = 0.09). In addition, during pediatric care, 36% (*n* = 18/50) of individuals received psychiatric follow-up, which declined to 26% (*n* = 13/50) in adult care, indicating a reduction in psychosocial support. These referrals were mostly made as part of routine multidisciplinary care due to adherence challenges or emotional difficulties associated with diabetes.

### Characteristics of individuals discontinuing follow-up after transition

The mean age of the 30 individuals who discontinued follow-up after their first adult endocrinology clinic visit at transition was 19.81 ± 2.20 years. The mean HbA1c during the last year of pediatric care was 8.84 ± 1.48%, and the average number of annual pediatric outpatient visits before transition was 3.24 ± 1.56.

Of these individuals, 53.8% were followed in tertiary care centers, 30.8% in secondary care centers, and 7.7% in primary care facilities, while 7.7% were not under regular follow-up currently. The most common factor for transfer to other centers was geographical proximity (56.4%), followed by relocation due to university education (17.9%). Healthcare system–related factors accounted for 15.4%, while individual preferences (10.3%) were reported at a lower rate. In this study, the term ‘discontinued follow-up’ refers specifically to individuals who did not return to our adult endocrinology clinic, even though many reported continuing care elsewhere, based on self-report rather than external medical records.

### Assessment of diabetes-related complications

**Figure 2:**
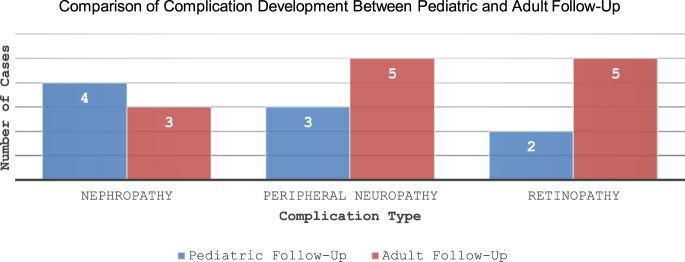
The frequency of nephropathy, peripheral neuropathy, and retinopathy is shown for pediatric (blue bars) and adult (red bars) follow-up periods. Data are presented as the number of cases affected in each category.

Among the 50 individuals who continued follow-up in adult care, 18% (*n* = 9/50) developed new chronic complications during the pediatric follow-up period, whereas 26% (*n* = 13/50) were found to have developed new complications during adult care (Fig. 2). The mean age of onset for microvascular complications was 15.94 ± 2.71 years during the pediatric period and 24 ± 3.57 years during adult follow-up. Diabetes-related complications were defined as the presence of any microvascular complication, including diabetic retinopathy (confirmed by ophthalmologic evaluation), nephropathy (defined by microalbuminuria), or peripheral neuropathy (based on clinical findings or nerve conduction studies).

### Therapeutic adjustments and insulin regimen modifications

**Figure 3:**
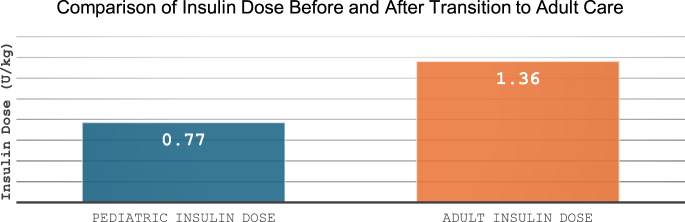
Mean total daily insulin dose (U/kg/day) is shown for pediatric follow-up (blue bar) and adult follow-up (red bar). Values indicate average insulin requirements per kilogram of body weight during the respective follow-up periods.

Before transition, 36% of individuals were on insulin pump therapy, while 64% were treated with multiple daily insulin injections (MDI). Following the transition, 54% (*n* = 27) of individuals underwent therapeutic adjustments. After the transition, the total daily insulin dose significantly increased, rising from 0.77 ± 0.20 U/kg/day in the pediatric period to 1.36 ± 0.54 U/kg/day in the adult period (*P* < 0.001) (Fig. 3). The most common adjustment was switching from U100 glargine to U300 glargine, accounting for 37% of cases (*n* = 10). This was followed by the addition of metformin in 19% (*n* = 5) of cases. Transitioning from detemir to U300 glargine occurred in 19% (*n* = 5), while 15% (*n* = 4) of individuals switched from MDI to insulin pump therapy. The least common adjustment was reverting from insulin pump therapy back to MDI, observed in 11% (*n* = 3) of cases. In addition, the proportion of basal insulin within the total insulin dose increased significantly from 44.65 ± 11.75% in pediatric care to 45.57 ± 9.97% in adult care (*P* = 0.0076).

## Discussion

The transition from pediatric to adult care for individuals with type 1 diabetes (T1D) represents a critical period concerning care adherence and metabolic control. This study assessed the effectiveness of a clinic in facilitating this transition, offering valuable insights into care continuity, glycemic control through HbA1c monitoring, complication development, and therapeutic regimen adjustments. Our findings contribute to a better understanding of the challenges encountered during the transition and reinforce the necessity of structured and individualized approaches, consistent with the existing literature.

Our study emphasizes the ongoing challenges in maintaining long-term follow-up after the transition from pediatric to adult diabetes care. Despite efforts to establish well-organized transition processes, a considerable proportion of individuals were lost to follow-up, while others eventually discontinued care after transfer. These patterns are consistent with previous reports highlighting the vulnerability of this transition phase. Garvey *et al.* and Pacaud *et al.* have drawn attention to how insufficient preparation and lack of support contribute to poor adherence during this critical period (9, 10). Similarly, studies by Cadario and Crowley *et al.* confirm that the complexity of transition processes can compromise continuity of care (4, 7). International guidelines, such as those by Gregory *et al.* in the ISPAD consensus, stress the importance of structured transition programs with individualized care plans to minimize the risk of disengagement (3). Our findings reinforce the need for proactive systems that ensure consistent support and follow-up, highlighting the importance of well-coordinated transition clinics and multidisciplinary engagement throughout the process.

In our clinic, individuals who did not continue follow-up in the adult endocrinology service were contacted by phone one by one, and reasons for discontinuation, current follow-up status, and – if applicable – reasons for changing centers were inquired. However, detailed long-term data from external centers could not be systematically obtained, which represents a limitation of this study.

Our findings showed that although most individuals discontinuing follow-up after transition continued their care in tertiary or secondary centers, a small but clinically relevant proportion remained without regular follow-up, consistent with previous reports of gaps in care continuity during transition (9, 11). In our cohort, geographical proximity and relocation due to university education were the leading reasons for transferring to other centers; while not specifically addressed in earlier studies, such findings align with prior reports emphasizing life-course changes, including residential and educational transitions, as determinants of continuity (12). Additional system-related barriers and, less frequently, dissatisfaction and communication issues were also observed, underscoring the need for structured transition models and improved patient–provider communication to enhance retention in adult care (13). For those who did not attend the first adult clinic appointment, the pediatric endocrinology team attempted to re-establish contact by phone to provide reminders, explore barriers, and encourage re-engagement; however, despite these efforts, a small but clinically relevant proportion remained disengaged from follow-up, underscoring the inherent challenges of sustaining care continuity during this period.

Our model does not aim to serve as a one-size-fits-all approach but rather reflects the real-world experience of a single tertiary center. This is particularly important for countries with large patient volumes and limited consultation time, where such an approach may provide practical benefits. In busy adult clinics, it can save time and allow physicians to directly obtain first-hand information from the care team about the challenges faced by individuals. A strength of this process was the active coordination between pediatric and adult teams at the time of transition, ensuring that the first appointment was scheduled and confirmed, together with the provision of detailed information about the patient. Furthermore, the systematic follow-up of individuals who discontinued care provided rare insights into the reasons for disengagement and their subsequent care pathways. The main limitation of our model was the absence of a formal joint clinic integrating both pediatric and adult endocrinologists, which may have limited opportunities for multidisciplinary decision-making and smoother adaptation. In our system, this limitation meant that patient adaptation was not fully supported. Nevertheless, in healthcare contexts similar to ours – particularly in middle-income and densely populated countries – our model may represent a pragmatic and resource-efficient strategy. In addition, limited psychosocial support and lack of systematically collected external long-term data represented further challenges. Nonetheless, our findings provide valuable insights into the real-life challenges of transition in a middle-income healthcare context, highlighting both strengths and areas for improvement that may guide future program development. Future multicenter studies with standardized outcomes will be required to determine whether such modifications can enhance long-term retention and clinical benefit.

In our study, the final annual visits during pediatric care were compared with the first-year follow-up assessments in adult care. In our model, after the transition decision was made during the joint pediatric–adult meeting and the patient was handed over, the adult endocrinology team directly contacted the individual to schedule the first appointment, and the pediatric team subsequently confirmed this contact with the patient. Although no formal joint clinic was implemented, this process ensured direct interaction with the adult team, supporting the classification of the model as a transition rather than merely a transfer. However, it is important to acknowledge the unique challenges of maintaining continuity of care in adolescents. This age group is particularly vulnerable to fluctuations in motivation, barriers to accessing healthcare services, and deficiencies in transition preparedness, all of which may adversely affect follow-up adherence. Therefore, future studies should not only focus on the last pediatric follow-up visits but also incorporate a comprehensive evaluation of the entire pre-transition period. Such an approach may facilitate the early identification of high-risk individuals and contribute to the development of more effective and individualized transition strategies. In our clinic, transition to adult care is typically planned after individuals with T1D begin their university education, and the timing is arranged based on their preferences and readiness. This may partly explain the higher average age of transition observed in our cohort compared to other pediatric centers.

### Metabolic control and nutrition support

Despite the transition from pediatric to adult care, our findings indicate that overall glycemic control remained stable, suggesting that the structured transition process may help prevent clinical deterioration. Similarly, Hatun *et al.* (14) demonstrated that structured pediatric care with early education, personalized targets, and integrated technology use contributes to better HbA1c outcomes before transition, which may facilitate smoother adaptation to adult care (14). This contrasts with previous reports, such as those by Nakhla *et al.*, which observed worsening metabolic control during transition periods (15), likely due to differences in care models or support structures. On the other hand, studies such as Holmes-Walker *et al.* highlight that dedicated transition programs can maintain glycemic stability and reduce hospitalization rates (16). The decline in nutrition support following transition in our cohort is noteworthy, as previous research (e.g., Hendricks *et al.*) suggests that reduced nutritional counseling may contribute to diminished self-care behaviors and, consequently, poorer metabolic outcomes (17). In this context, the slight increase in BMI we observed, although not statistically significant, underscores the need to monitor weight trends closely. Prior studies have linked early weight gain and prolonged diabetes duration to BMI increases in this population (5, 18). These findings reinforce the critical importance of integrated dietary and psychosocial support throughout the transition period.

### Psychiatric follow-up and the transition process

Our findings reveal a decline in psychiatric follow-up from the pediatric to adult care period, underscoring the difficulty of sustaining psychosocial support throughout the transition. Consistent with our results, the ISPAD guidelines by Gregory *et al.* highlight the need for ongoing psychosocial care to ensure a smooth and effective transition (3). Weissberg-Benchell *et al.* further emphasize that inadequate psychosocial support during this period may lead to adverse outcomes, such as reduced treatment adherence and heightened emotional distress (5). To address this gap, strengthening multidisciplinary coordination becomes essential. Van Walleghem *et al.* demonstrated the effectiveness of a systems navigator model in maintaining continuity of care, particularly by supporting psychosocial needs during the transition phase (19). These observations collectively stress the importance of integrating psychological services into structured transition programs.

### Complication development

The observed increase in complication rates from pediatric to adult care in our cohort likely reflects the cumulative burden of long-standing diabetes. This finding aligns with previous studies by Schwartz and Eisenberg, which identified diabetes duration as a major predictor of microvascular and macrovascular complications (8, 20). Our results are further supported by the DCCT/EDIC study (21), which similarly demonstrated that longer disease duration significantly increases the risk of complications (21). These findings highlight the critical role of early preventive strategies and longitudinal follow-up to mitigate long-term risks in individuals with T1D.

### Therapeutic adjustments and insulin dose

In our study, the need for therapeutic modifications in more than half of the participants highlights that the transition period should not be regarded merely as a shift in care setting but as a critical opportunity to re-evaluate evolving metabolic demands. Physiological changes such as the completion of puberty, increased insulin resistance, and early adulthood weight gain likely contributed to the increased insulin requirements observed in this period. As emphasized by Peters *et al.* (13), reassessing basal insulin needs during the transition is essential to ensure optimal glycemic control. In line with this, our study also demonstrated a significant rise in the proportion of basal insulin use (13). One of the most frequently implemented changes was the switch from U100 to U300 glargine, which not only improved glycemic stability but also enhanced patient satisfaction. This is consistent with findings by Crowley *et al.* (7), who reported that individualized therapeutic adjustments during this stage positively influence adherence and metabolic outcomes. Altogether, these observations underscore the importance of restructuring diabetes management plans in accordance with the dynamic physiological and psychosocial needs of emerging adults during the transition phase.

### The role of transition clinics and future recommendations

Our findings underscore the crucial role of structured transition clinics in facilitating the transfer of individuals with type 1 diabetes (T1D) to adult care while promoting continuity of follow-up. Elements such as educational interventions, individualized transition planning, and psychosocial support are key components of successful transition models. The ISPAD guidelines, as emphasized by Gregory *et al.*, advocate for personalized transition protocols to improve clinical outcomes (3). Similarly, Pacaud *et al.* (6) and de Beaufort *et al.* (22) have shown that structured programs offering regular follow-up and tailored support significantly enhance adaptation to adult services (6, 22). These strategies support glycemic control while concurrently addressing the psychological and educational needs of adolescents and young adults with T1D.

Looking ahead, future research should include larger cohorts and adopt prospective study designs, including long-term observational follow-up and standardized surveys. Such approaches will allow for a deeper understanding of the barriers to effective transition and aid in the development of more robust, evidence-based transition strategies.

In addition, future studies should incorporate the perspectives of young individuals with T1D regarding the transition process to better understand psychosocial barriers and individual needs. Furthermore, future prospective studies should aim to investigate the reasons behind non-engagement in adult follow-up among those who never attended post-transition, as this remains a critical challenge to ensuring care continuity. Finally, our study did not include data on emergency department visits or hospitalizations after transition, which represents a limitation, since such outcomes could provide important insights into acute complications and healthcare utilization patterns during this vulnerable period. Moreover, socioeconomic data were not routinely available in clinical records and, therefore, could not be analyzed, which represents an additional limitation of our study.

## Conclusion

This study assesses the challenges faced by individuals with T1D during the transition from pediatric to adult care, focusing on follow-up continuity, metabolic control, complication development, and treatment modifications. Our findings underscore the importance of maintaining follow-up and multidisciplinary support to ensure long-term health outcomes. Support from dietitians and psychiatrists during the transition plays a vital role in maintaining metabolic control and preventing complications. The reduction in psychiatric follow-up highlights the need for enhanced support and monitoring strategies. Furthermore, the positive impact of treatment modifications on glycemic control demonstrates the importance of personalized, regularly updated treatment plans. In this context, by documenting both strengths and shortcomings, our study provides rare real-world evidence from a middle-income healthcare setting, offering insights that may inform future transition program development. Our results support the need for implementing transition clinics and personalized transition plans to make the process more effective and sustainable. Future research should thoroughly explore the transition process using larger populations and prospective data collection. This will help individuals with T1D successfully adapt to adult care and improve their long-term health outcomes.

## Declaration of interest

The authors declare no conflict of interest. Samim Özen is a Senior Editor of *Endocrine Connections*. Samim Özen was not involved in the review or editorial process for this paper, on which he is listed as an author.

## Funding

This work did not receive any specific grant from any funding agency in the public, commercial, or not-for-profit sectors.

## Author contribution statement

Uğur Cem Yılmaz: conceptualization, formal analysis, investigation, methodology, visualization, writing – original draft (lead). Melih Bektaş: data curation, resources. Yiğit Özel: data curation. Günay Demir: data curation, resources, statistical analysis. Deniz Özalp Kızılay: writing – review & editing, visualization. Şükran Darcan: data curation, resources. Samim Özen: supervision, writing – review & editing. Vildan Özkan Derviş: data curation, resources. Ilgın Yıldırım Şimşir: supervision, data curation, resources. Damla Gökşen: project administration, resources, supervision (lead), writing – review & editing (lead).

## Data availability

The data supporting the findings of this study are available from the corresponding author upon reasonable request.

## Scope and limitations

This study is limited to the transition process conducted at our center, and the generalizability of the results may be restricted. In addition, the retrospective nature of the data may result in certain information gaps.

## Ethical approval

Ethical approval was obtained from the Ege University Clinical Research Ethics Committee (Approval No.: 25-4T/3). Written informed consent was obtained from all participants. All procedures complied with principles of confidentiality and data protection, and participant data were anonymized before analysis.
